# Identified Gefitinib Metabolism-Related lncRNAs can be Applied to Predict Prognosis, Tumor Microenvironment, and Drug Sensitivity in Non-Small Cell Lung Cancer

**DOI:** 10.3389/fonc.2022.939021

**Published:** 2022-07-01

**Authors:** Weilong Ye, Zhengguo Wu, Pengbo Gao, Jianhao Kang, Yue Xu, Chuzhong Wei, Ming Zhang, Xiao Zhu

**Affiliations:** ^1^ School of Laboratory Medicine and Biological Engineering, Hangzhou Medical College, Hangzhou, China; ^2^ Computational Oncology Laboratory, Guangdong Medical University, Zhanjiang, China; ^3^ Department of Thoracic Surgery, Yantian District People’s Hospital, Shenzhen, China; ^4^ Department of Physical Medicine and Rehabilitation, Zibo Central Hospital, Zibo, China

**Keywords:** drug sensitivity, gefitinib, lncRNAs, non-small cell lung cancer, P450

## Abstract

Gefitinib has shown promising efficacy in the treatment of patients with locally advanced or metastatic EGFR-mutated non-small cell lung cancer (NSCLC). Molecular biomarkers for gefitinib metabolism-related lncRNAs have not yet been elucidated. Here, we downloaded relevant genes and matched them to relevant lncRNAs. We then used univariate, LASSO, and multivariate regression to screen for significant genes to construct prognostic models. We investigated TME and drug sensitivity by risk score data. All lncRNAs with differential expression were selected for GO/KEGG analysis. Imvigor210 cohort was used to validate the value of the prognostic model. Finally, we performed a stemness indices difference analysis. lncRNA-constructed prognostic models were significant in the high-risk and low-risk subgroups. Immune pathways were identified in both groups at low risk. The higher the risk score the greater the value of exclusion, MDSC, and CAF. PRRophetic algorithm screened a total of 58 compounds. In conclusion, the prognostic model we constructed can accurately predict OS in NSCLC patients. Two groups of low-risk immune pathways are beneficial to patients. Gefitinib metabolism was again validated to be related to cytochrome P450 and lipid metabolism. Finally, drugs that might be used to treat NSCLC patients were screened.

## Introduction

Lung cancer, one of the most common cancers, accounts for a quarter of all cancer deaths. Non-small cell lung cancer (NSCLC) is the most common histologic subtype. It accounts for about 85% of whole lung cancers (1). In China, the incidence and mortality of lung cancer list first among malignant tumors (2). Among them, NSCLC accounts for approximately 80%-85% of all lung cancers (3, 4). Conventional chemotherapy was one of the first forms of treatment for cancer, but it has not improved the 5-year survival rate. When conventional chemotherapy was used to kill the tumor cells, it also caused damage to normal tissues and organs in the body (5–7). With the discovery of tumor driver genes, targeted therapy for lung cancer has set up new pathways of treatment with low toxicity and high selectivity. In 2009, Iressa Pan-Asia Study (IPASS) showed that patients remedied with gefitinib had longer progression-free survival than those remedied with carboplatin-paclitaxel in a subgroup of 261 patients with epidermal growth factor receptor (EGFR) mutations (8). It competes with the EGFR-TK catalytic region to block downstream pathways and inhibit tumor growth, metastasis, and angiogenesis (9–11). Gefitinib is suitable for locally advanced or metastatic NSCLC with an EGFR-sensitive mutation (9, 12, 13). Gefitinib is broadly metabolized in the liver by cytochrome P450 to five metabolites, primarily by CYP3A4 and, to a smaller extent, by CYP3A5 and CYP2D6 (14).

In the process of receiving treatment, patient response to treatment options usually requires assessment of the lesion with the assistance of imaging. However, conventional imagery cannot identify the evolution of the tumor during treatment, or provide further indication of the patient’s prognosis (15). The use of tissue biopsy as a method of assessment can cause great pain to the patient and also increase the potential risk of tumor metastasis (16, 17). Currently, the evidence suggests that the detection and application of molecular biomarkers could provide prognostic value (18). So far, no studies have been conducted to predict patient prognosis with gefitinib metabolism-related lncRNA gene signatures (GMLncSig), and molecular biomarker studies are essential for prognosis prediction and determination of personalized therapy.

LncRNAs are non-coding RNA over 200 nucleotides in length and have been shown to engage in a wide range of biological and cellular functions (19). One of the studies performed whole transcriptome RNA sequencing in patients with different types of melanoma immune cells and identified 27,625 lncRNAs, some of which were significantly associated with cancerous status (20–22). A growing number of studies have shown that lncRNAs are closely associated with tumorigenesis, progression, prognosis, susceptibility, and drug resistance (23, 24). Therefore, there is a necessity to include lncRNAs in preclinical models to allow the development of prognostic biomarkers.

The tumor microenvironment (TME) is a multifaceted cytosolic environment that both limits tumor development (25–27) and plays a key role in tumor progression and therapeutic response (28–31). In lung adenocarcinoma (LUAD), they evaluated the correlation between the m6A-related lncRNA model and immunotherapy biomarkers, and the m6A-based classifier index can be used as a predictor of TIDE and TMB (32). To date, the broad landscape of TME in immune cell-infiltrated has not been elucidated.

In this study, we aimed to screen out gefitinib metabolism-related lncRNAs and explore their prognostic effects, immune microenvironment, and drug sensitivity in NSCLC.

## Method

### Download the Databases of Related Genes and Patient Cohorts

In this study, we downloaded seven genes about the gefitinib drug pathway from Rat Genome Database (RGD), (https://rgd.mcw.edu/; PW:0000869) (33). Gene sets “COLDREN_GEFITINIB_RESISTANCE_DN” (n=230) and “COLDREN_GEFITINIB_RESISTANCE_UP” (n=86) belong to Molecular Signatures Database (MSigDB) (34). We also obtained 92 gefitinib metabolism-related genes in the GeneCards database. The Human Gene Database (https://www.genecards.org/), is a source for gene-related information, which includes a great deal of gene-centric records from 150 web sources (35). The information from the three databases was combined and duplicate genes were excluded, resulting in 394 genes. We obtained gene expression, clinicopathological and prognostic data from The Cancer Genome Atlas (TCGA) for 494 patients with NSCLC. Patient data from the IMVigor210 clinical trial were downloaded from the European Genome-Phenome Archive. Finally, we used the LIMMA package with the R software (version 4.0.2; https://cran.r-project.org/) to obtain all GMLncSig with the gene sets and visualized them with a Sankey diagram.

### Construction of the lncRNAs-Related Prognostic Model

First, we screened the entire cohort for meaningful genes using univariate Cox regression analysis. 494 NSCLC patients were randomly assigned to a training cohort (n=330) and a testing cohort (n=164). Then, we conducted a univariate Cox regression analysis on the training subgroup to establish the correlation between lncRNA-related and overall survival (OS) in NSCLC patients and selected the genes dramatically associated with OS as prognosis-related genes (P<0.05). Given the impact of multicollinearity between the variables, we performed a least absolute shrinkage and selection operator (LASSO) Cox regression analysis (36–40). Finally, multivariate Cox regression analysis was used to construct prognostic features based on the potential candidate lncRNAs derived from the above screening. Patients’ risk scores were estimated using the following formula:


$$GMLncSig=\sum_{i=1}^{n}Coef\left(i\right)\times x\left(i\right)$$

where Coef(i) and x(i) denote the regression coefficients estimated by multivariate Cox regression analysis and the GMLncSig expression values, respectively. We divided the NSCLC patients in the training cohort into high-risk and low-risk subgroups using the median risk score as the cut-off value and analyzed the OS of the two subgroups using Kaplan-Meier survival curves. Next, we evaluated the reliability and robustness of the prognostic risk score using the testing cohort and the entire cohort. We also used univariate and multivariate Cox regression analysis to assess the prognostic of other clinicopathological factors such as gender, age, American Joint Committee on Cancer (AJCC) stages, race, primary tumor (T), and regional lymph nodes (N), and distant metastasis (M). Next, we use receiver operating characteristic (ROC) (41) curves and concordance index (C-index) curves to evaluate the predictive effect of risk scores. Nomogram was used to construct a visual predictive model. For the sake of rigor, we also validated the model for clinical subgroup data. Principal component analysis (PCA) was used to evaluate the distinction of related genes in the high-risk and low-risk subgroups.

### Gene Ontology/Kyoto Encyclopedia of Genes and Genomes Enrichment Analyses

To further investigate the GMLncSig, we compared all lncRNA expressions of patients in high and low-risk subgroups and retrieved lncRNAs that were significantly different (p<0.05). Later, all differential lncRNAs were enrolled in the GO/KEGG pathway enrichment analyses. Gene Ontology (GO) and Kyoto Encyclopedia of Genes and Genomes (KEGG) pathway enrichment analyses with the R package were performed. A Fisher exact test was used and those with a false discovery rate (FDR) corrected p-value less than 0.05 were regarded as significant indicators. GO and KEGG are both based on the Database for Annotation, Visualization, and Integrated Discovery (DAVID) (https://david.ncifcrf.gov/).

### The Tumor Microenvironment Analyses of GMLncSigs

Immune-related lncRNAs (correlation (|cor|) >0.6, p<0.001) and GMLncRNAs (|cor| >0.4, p<0.001) were identified using Spearman correlation analysis between mRNAs and lncRNAs. GSVA is a gene-set enrichment method that estimates changes in the pathway and biological process activity in a sample population without supervision (42). We used “GSVA” packages for R to analyze the gene sets, setting the significance threshold to an adjusted P-value < 0-05. We compared the Tumor Mutation Burden (TMB) of the high-risk and low-risk subgroups and also visualized them using the survival curve. TMB was defined as the total number of somatic gene coding errors, base substitutions, gene insertions, or deletion errors detected per million bases (43). High TMB values predict better immunotherapy outcomes for participants (44). Then, we analyzed tumor immune escape and immunotherapy in related lncRNA. The Tumor Immune dysfunction and Exclusion (TIDE) algorithm, a computational method for modeling the two main mechanisms of tumor immune evasion induces T-cell dysfunction in tumors with high infiltration of cytotoxic T lymphocytes (CTL) and protects against T-cell infiltration in tumors at low CTL levels (45). The data from the TIDE web platform (http://tide.dfci.harvard.edu/) (46). In addition, we have analyzed other immune biomarkers or cells, including Microsatellite Instability (MSI) score, Merck18, a cluster of differentiation 274 (CD274), IFGN, CD8 (47–49), Myeloid-derived suppressor cell (MDSC) (50), cancer-associated fibroblasts (CAF) (51) and tumor-associated macrophages M2 (TAMM2) (52).

### Exploration of Drug Resistance and Screening

We used the pRRophetic algorithm based on Genomics of Drug Sensitivity in Cancer (GDSC) semi-inhibitory concentrations to estimate the therapeutic response of the samples to identify potential drugs for our lncRNA model to treat NSCLC patients (53).

### Correlation With Immunotherapy Response in IMvigor210 Cohort

To validate the prognostic value of the lncRNA model, we used the IMvigor 210 model for the evaluation. IMvigor210 Study to evaluate the efficacy and safety of the Programmed cell death ligand 1 (PD-L1) targeted antibody atezolizumab in platinum-treated patients with the locally advanced or metastatic uroepithelial disease (54, 55). IMvigor210CoreBiologies package was used to download the data. First, we matched the genes obtained from the LASSO regression analysis to those in the IMvigor210 model. The corresponding risk score for each patient in the IMvigor210 cohort was then calculated by the same risk score formula and patients were classified into high-risk and low-risk subgroups.

### Calculation With the Stemness Indices (mRNAsi)

Derived from the Progenitor Cell Biology Consortium (PCBC) database, the characteristics of stemness were recognized by the one-class logistic regression (OCLR) algorithm (56). The higher the value of mRNAsi, the greater the tumor dedifferentiation and stemness (57). Based on the OCLR algorithm, we first calculated the stemness index (mRNAsi) for each patient in the TCGA-NSCLC cohort using RNA-seq data. Then, we ranked each patient based on the mRNAsi score and tested the relationship between this index and clinicopathological characteristics. The whole research process is shown in the chart (**Figure S1**).

## Result

### Identification of Gefitinib Metabolism-Associated lncRNA

We ran the limma package with the R software and identified the gefitinib metabolism-related lncRNAs. Then, we use the ggalluvial R package to visualize the results (**Figure S2**). Univariate Cox regression analysis showed that 56 lncRNAs were significant (pvalue<0.05) and the expressions of AC004704.1, AL162632.3, LINC00707, and AL161668.1 were significant with a p-value less than 0.001 (**Table S1**)

### Identification of 13 GMLncSigs in a Training Cohort of NSCLC Patients

**Figure 1:**
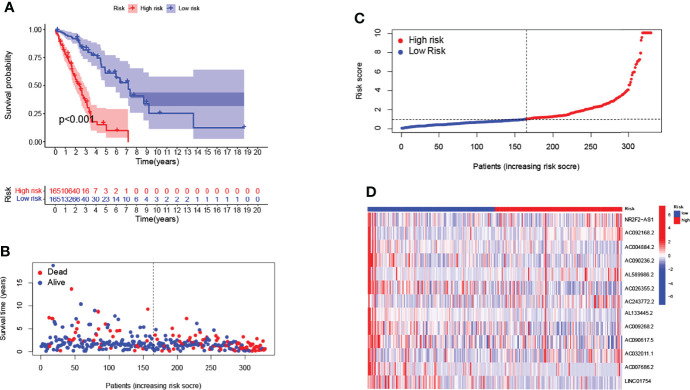
Construction of 13-lncRNA signature in the training cohort. Longer survival time by Kaplan-Meier test for patients in the low-risk group **(A)**. Survival status and risk scores are shown for each case **(B, C)**. Heatmap showing the expression of GMLncSigs. Rising risk score from blue to red. AL133445.2, LINC01754, and AC090236.2 are lowly expressed in the high-risk subgroup and highly expressed in the low-risk subgroup **(D)**.

We randomized all patients (n=494) into the training cohort (n=330) and testing cohort (n=164), with no significant differences in clinical characteristics (Pvalue>0.05). We next analyzed the risk characteristics for predicting the prognosis in the training cohort. We screened the lncRNAs using univariate Cox regression analysis and lasso Cox regression analysis, the results were shown in **Table S2** and **Figure S3** respectively. Finally, 13 GMLncSigs were identified using multivariate Cox regression analysis (**Table S3**). We then calculated the risk score for each patient in the training cohort and divided them into high-risk (n=165) and low-risk (n=165) subgroups based on the median risk score. Kaplan-Meier survival curve analysis showed that the overall survival time of NSCLC patients in the high-risk subgroup was significantly lower than that of NSCLC patients in the low-risk subgroup(p<0.001) (**Figure 1A**). The risk score distribution of patients in the high- and low-risk subgroups showed that the survival rate of the high-risk subgroup was significantly lower than the low-risk subgroup (**Figures 1B, C**). The heatmap showed the differential expression of 13 risk-related lncRNAs in the high-risk and low-risk subgroups, where it can be seen that AL133445.2, LINC01754, and AC090236.2 are lowly expressed in the high-risk subgroup and highly expressed in the low-risk subgroup (**Figure 1D**).

### Validation of Prognostic Risk Signature in the Testing Cohort and Entire Cohort

We validated the robustness and accuracy of the prognostic risk signature in the testing cohort and the entire cohort. Prognostic risk scores were calculated for testing (n=164) and entire cohort (n=494) based on prognostic risk signature, and patients were classified into high-risk subgroup (testing cohort n=90; entire cohort n=255) and low-risk subgroup (testing cohort n=74; entire cohort n=239) based on median critical values. Kaplan-Meier survival curve analysis showed that overall survival was significantly shorter in high-risk patients than in low-risk patients for both the testing cohort (P=0.04; **Figure S4A**) and the entire cohort (P<0.001; **Figure S4B**). The distribution of risk scores and survival status in the testing cohort and the entire cohort were shown in **Figures S4C-F**. The heatmap shows the differential expression of 13 risk-related lncRNAs in the high-risk and low-risk subgroups (**Figures S5A, B**). AL133445.2, LINC01754, and AC090236.2 were lowly expressed in the high-risk subgroup and highly expressed in the low-risk subgroup, and the results were consistent with the training subgroup. Overall, our findings showed that the prognostic risk signature accurately predicted the survival outcome of NSCLC patients.

### Relationship Between GMLncRNAs and Clinicopathological Parameters of NSCLC Patients

**Figure 2:**
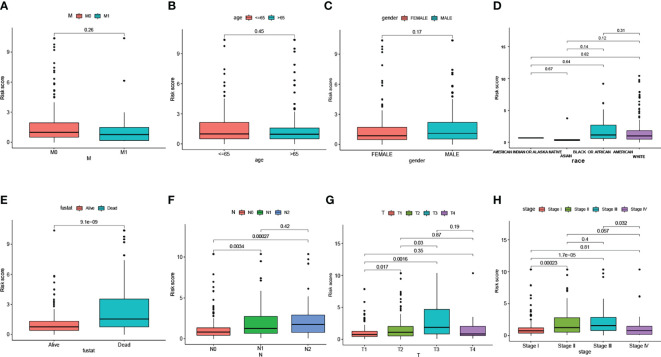
The scatter diagram showed the correlation between clinicopathological factors and risk scores. Which include M stage **(A)**, age **(B)**, gender **(C)**, race **(D)**, fustat **(E)**, N stage **(F)**, T stage **(G)**, and AJCC stage **(H)**.

We visualized the relationship between clinicopathological factors and risk scores. It can be seen that M (p=0.26), age (p=0.45), gender (p=0.17), race (p>0.05) and risk score were not correlated (**Figure 2A–D**). In addition, early-stage tumors were significantly associated with the low-risk subgroup, while late-stage tumors were significantly associated with the high-risk subgroup (**Figures 2E–H**).

### Analysis of Patient Clinical Independent Prognostic Models

**Figure 3:**
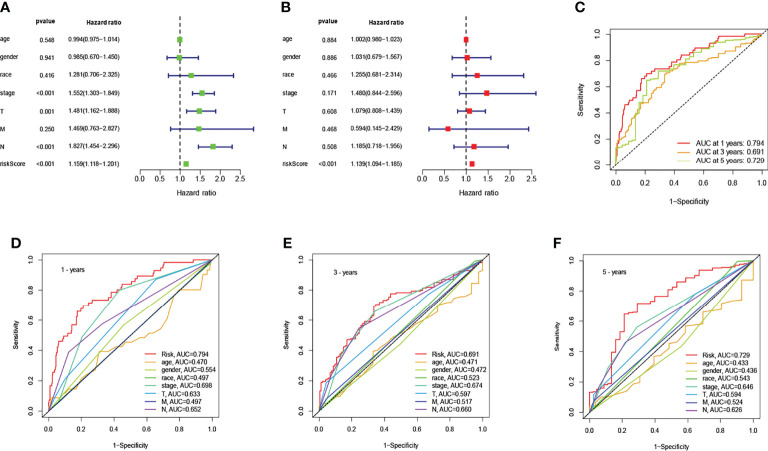
Association of risk scores with clinicopathological factors. Univariate **(A)** and multivariate **(B)** Cox regression analysis of OS in the entire cohort (n = 494). The data are expressed as hazard ratio (HR) ± 95% confidence interval [CI]. The ROC of the optimal model for 1-years, 3-years, and 5-years shows AUC values of 0.794, 0.691, and 0.729, respectively **(C)**. Comparison of the ROC curves at 1-years **(D)**, 3-years **(E)**, and 5-years **(F)** with other common clinical features showed the advantage of risk scoring.

To identify an independent prognostic factor, we performed univariate and multivariate Cox regression analyses to identify clinicopathological factors, including age, gender, race, survival status, AJCC stage, T stage, N stage, and M stage. In the entire cohort, univariate Cox regression analysis showed that AJCC stage, T stage, N stage, and risk score were significantly associated with OS (**Figure 3A**). Multivariate Cox regression analysis showed that only risk score was significantly associated with OS (**Figure 3B**). Next, we assessed the quality of the patient’s clinically independent prognostic model using ROC curves. The ROC curves showed that clinicopathological factors had significant prognostic value in determining 1, 3, and 5-year survival rates in NSCLC patients (1 year AUC=0.794, 3 year AUC=0.691, 5 year AUC=0.729, **Figure 3C**). In determining the 1-year survival of NSCLC patients, risk scores (AUC=0.794), gender (AUC=0.554), stage (AUC=0.698), T (AUC=0.633), and N (AUC=0.652) were significant (**Figure 3D**). 3-year and 5-year survival rates are shown in **Figures 3E, F**. Overall, the risk score was a better predictor than other factors, and other factors can be used as a reference point.

**Figure 4:**
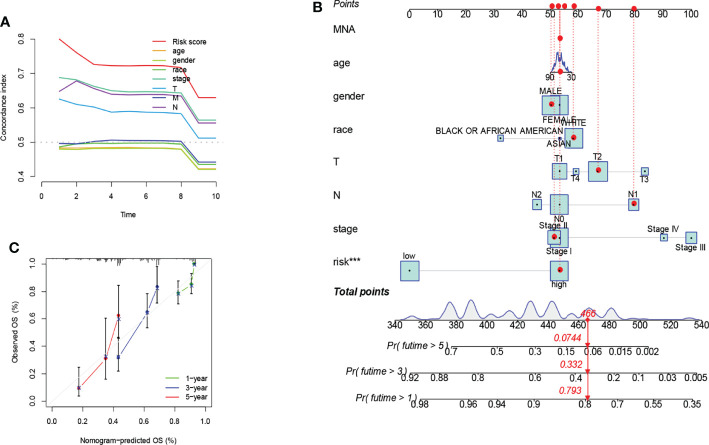
Prognostic model constructed by risk score and clinicopathological factors. Significant prognostic effects were shown in the C-index curve **(A)**. A nomogram was created to predict the survival rate of NSCLC patients. A vertical line is drawn from the variable value to the shaft labeled “point”. Points were then calculated across all variables. The total number of points on the bottom scale corresponds to 1-years, 3-years, and 5-years survival rates "***" means that P value <0.001. **(B)**. A calibration curve for the overall survival column line plot model across the cohort. The white diagonal line is the ideal column line plot, and the green, blue and red lines indicate the observed column line plots for 1-years, 3-years, and 5-years, respectively **(C)**.

The C-index curve showed significant prognostic effects for risk score, AJCC stage, N stage, and T stage (**Figure 4A**). That was consistent with the results of the ROC curve. Considering clinicopathological covariates, we chose a nomogram to construct an intuitive prediction model. Based on univariate and multivariate Cox regression analysis, we constructed a nomogram to predict patients’ OS at 1,3, and 5 years (**Figure 4B**). Calibration plots for 1-year, 5-year, and 10-year OS were well predicted compared to the ideal model in the entire cohort (**Figure 4C**).

### Model Validation of Clinical Subgroup Data

**Figure 5:**
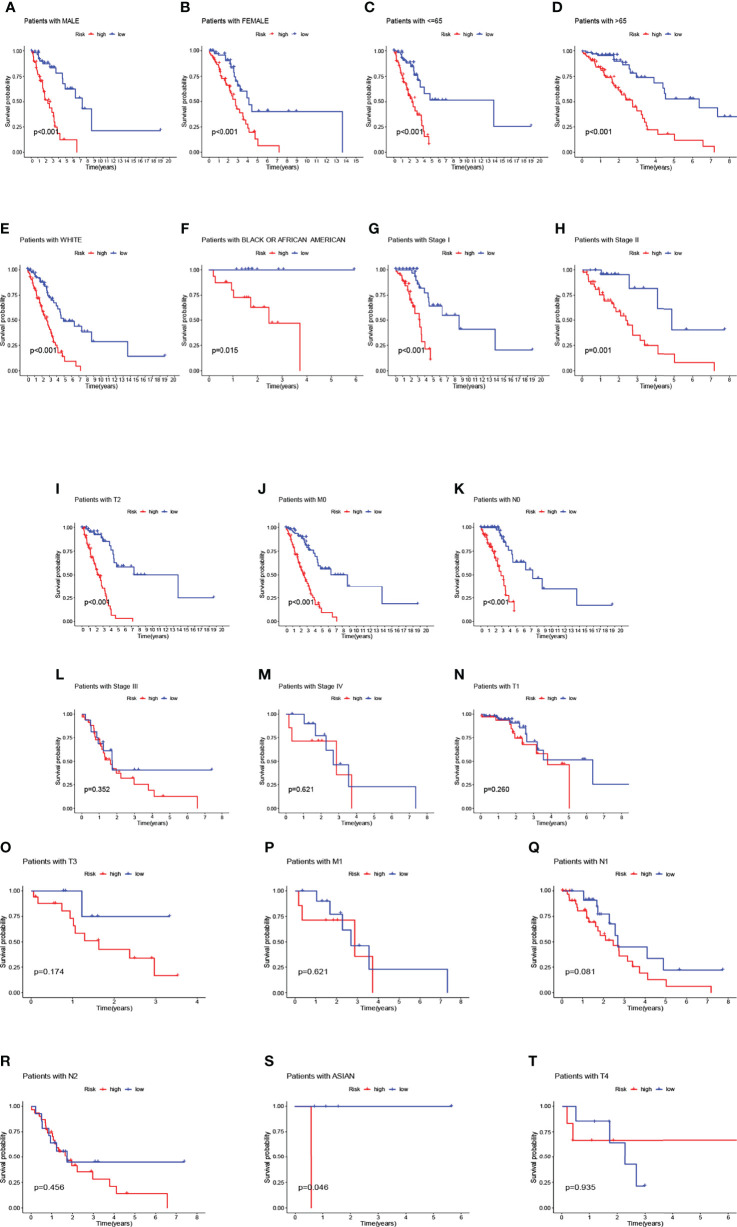
Overall survival of patients with high- and low-risk NSCLC stratified by clinicopathological parameters. Kaplan-Meier survival curves showed the OS rates of high-and low-risk subgroups stratified by male **(A)** and female **(B)**, age ≤ 65 **(C)** and > 65 **(D)**, race-white **(E)**, race-black or African American **(F)**, AJCC stages I-IV, T1-T4 stage, M0-M1 stage, N0-N2 stage, race-Asian **(G–T)**.

We observed that compared to patients with low risk scores, male patients (p<0.001), female patients (p<0.001) (**Figures 5A, B**), patients younger than 65 years (p<0.001), patients older than 65 years (p<0.001) (**Figures 5C, D**), race-white (p<0.001), patients with black or African American (p=0.015) (**Figures 5E, F**), patients with stage I (p<0.001) and II (p=0.001) (**Figures 5G, H**), patients with T2 (p< 0.001) (**Figure 5I**), patients with M0 (p<0.001) (**Figure 5J**), patients with N0 (p<0.001) (**Figure 5K**) had significantly shorter overall survival rates. However, in the high-risk and low-risk subgroups, OS rates for patients with stage III (p=0.352) and IV (p=0.621) (**Figures 5L, M**), patients with T1 (p=0.260) and T3 (p=0.174) (**Figures 5N, O**), patients with M1 (p=0.621) (**Figure 5P**), patients with N1 (p=0.081) and N2 (p=0.456) (**Figures 5Q, R**) were not related to risk scores. Patients with Asian and patients with T4 were discarded due to insufficient data (**Figures 5S, T**). We used principal component analysis (PCA) to visualize the differentiation of coding genes, non-coding genes, and all genes (mRNA and lncRNA) for all patients. It can be seen that the risk-related lncRNA distinction was significant (**Figures S6A–C**).

### Differential Expression Analysis of lncRNAs in GO/KEGG

**Figure 6:**
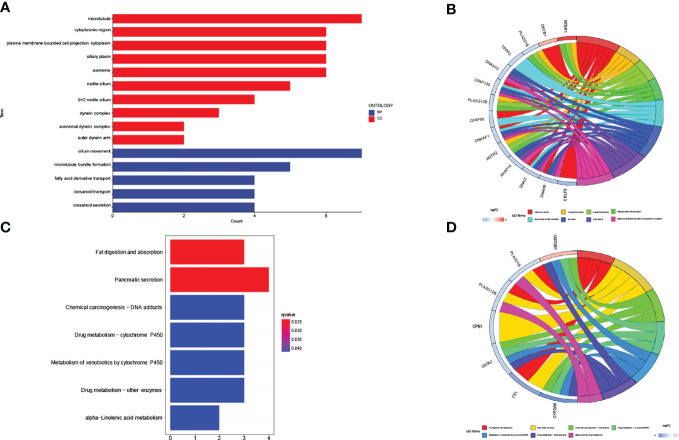
GO/KEGG pathway for all differential lncRNAs (n=153) between high-risk subgroup and low-risk subgroup. **(A, B)** The result of GO pathway analysis. **(C, D)** The result of KEGG pathway analysis.

We compared the lncRNA expression between patients in the low-risk subgroup and those in the high-risk subgroup and extracted meaningful (p<0.05) lncRNAs. GO pathway enrichment results showed up to 7 lncRNAs were closely associated with the pathways, such as (GO: 0003341: cilium movement, GO: 0005874: microtubule). We also need to pay attention to GO:0031514 (motile cilium), which may be more closely related to lung cancer (**Figure 6A**). We visualized representative lncRNAs and GO terms in **Figure 6B**. The KEGG pathway results showed differences in genes associated with lipid metabolism, such as (hsa04975: Fat digestion and absorption, hsa00592: alpha-Linolenic acid metabolism) and also with Drug metabolism, for example (hsa00982: Drug metabolism - cytochrome P450, hsa00980: Metabolism of xenobiotics by cytochrome P450, hsa00983: Drug metabolism - other enzymes) (**Figure 6C**). We visualized representative lncRNAs and KEGG terms in **Figure 6D**.

### Study on Immune Pathway Enrichments of GMLncSigs

The 13 immune function pathways in the high and low-risk subgroups were shown by a heat map. In the training cohort, Type_II_IFN_Reponse, HLA, and T_cell_co-stimulation can be seen to be highly expressed in the low-risk subgroup and lowly expressed in the high-risk subgroup (**Figure S7A**), indicating that they are low-risk pathways. In the testing and entire cohort, the immune function pathway expression profile was consistent with that of the training cohort (**Figures S7B, C**).

### Differential Analysis of TMB of GMLncSigs

**Figure 7:**
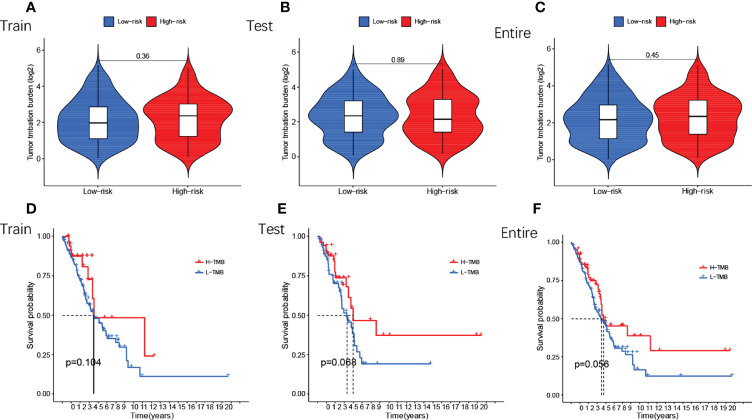
Differential analysis of TMB of GMLncSigs. The box plot and the survival curve revealed that there was no difference in TMB between high-risk and low-risk subgroups **(A)** in the training cohort **(B)** in the testing cohort **(C)** in the entire cohort. Another survival curve revealed that survival time varies among TMB-risk scores **(D)** in the training cohort **(E)** in the testing cohort **(F)** in the entire cohort.

**Figure 8:**
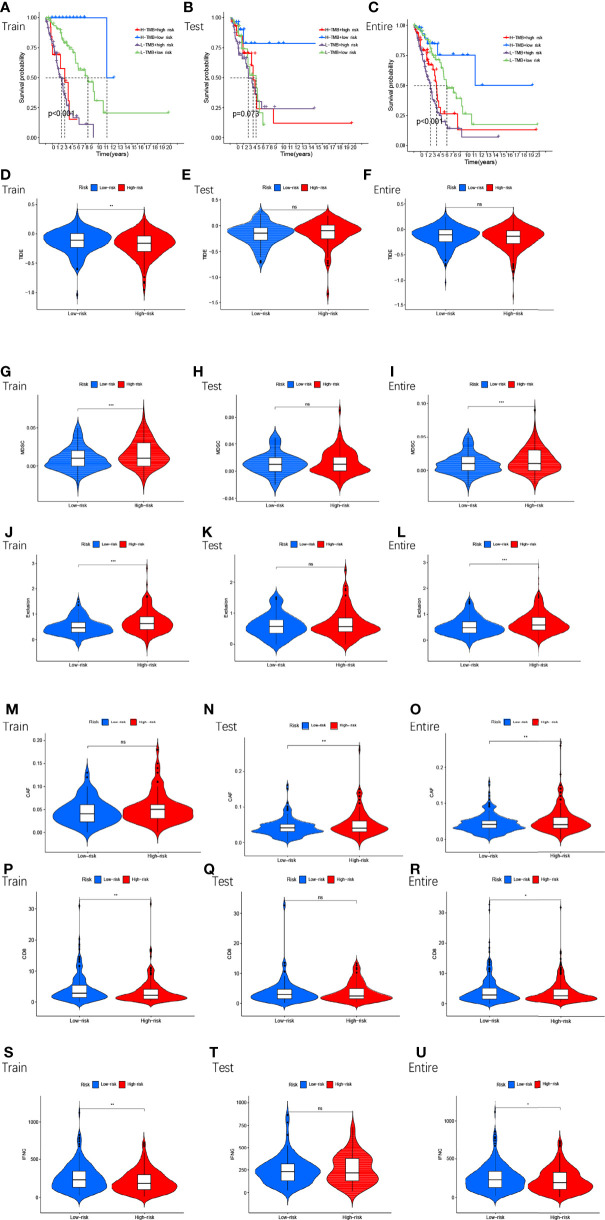
The survival of TMB+ risk subgroup in train cohort **(A),** test cohort **(B)**, entire cohort **(C)**. Tumor microenvironment results based on GMLncSigs. Analysis of the differences among TIDE **(D–F)**, MDSC **(G–I)**, exclusion **(J–L)**, CAF **(M–O)**, CD8 **(P–R)**, and IFNG **(S–U)** in different risk subgroups. "*" means that P value<0.05, "**" means that P value<0.01, "***" means that P value<0.001. "ns" means no significance.

Next, we compared TMB between the high and low-risk subgroups and found no difference between the training cohort (p=0.36) (**Figure 7A**), testing cohort (p=0.89) (**Figure 7B**), and entire cohort (p=0.45) (**Figure 7C**) between the high and low-risk subgroups (p>0.05). The results of the TMB survival curves for the training cohort (p=0.104) (**Figure 7D**), testing cohort (p=0.068) (**Figure 7E**), and entire cohort (p=0.056) (**Figure 7F**) were as we expected, and they were not statistically significant. To visually analyze the relationship between TMB, high-low risk subgroups, and survival, we made a survival curve. In the entire cohort (p<0.001), it can be seen that the H-TMB+low risk subgroup had the longest survival while the L-TMB+high risk subgroup had the shortest survival (**Figure 8A**). The training and testing cohorts are shown in **Figures 8B, C**.

### Analysis of Tumor Immune Escape and Immunotherapy of GMLncSigs

**Figure 9:**
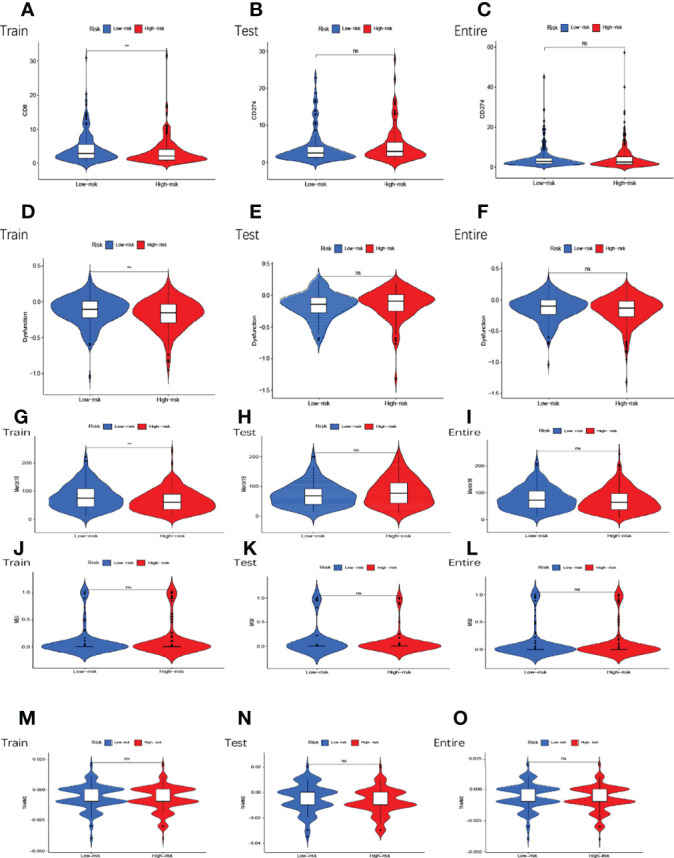
Tumor microenvironment results based on GMLncSigs. Analysis of the differences among CD274 **(A–C)**, dysfunction **(D–F)**, Merck18 **(G–I)**, MSI **(J–L)**, and TAMM2 **(M–O)** in different risk subgroups. "*" means that P value<0.05, "**" means that P value<0.01, "***" means that P value<0.001. "ns" means no significance.

In the high and low-risk subgroups, we analyzed tumor immune escape and immunotherapy for the related lncRNAs. It is worth mentioning first that the combined TIDE scores of the training cohort were lower in the high-risk subgroup and higher in the low-risk subgroup, and statistically significant between the high and low-risk subgroups (p<0.01,**) (**Figure 8D**). This result was unexpected. But it does not make sense in the entire cohort and the testing cohort (**Figures 8E, F**). MDSC were statistically different between high and low-risk subgroups (**Figures 8G–I**). The exclusion was statistically different between the high and low-risk subgroups (**Figures 8J–L**). CAF are statistically different between the high and low-risk subgroups (**Figures 8M–O**). The remaining immune markers such as CD8 (**Figures 8P–R**), IFNG (**Figures 8S–U**), CD274 (**Figures 9A–C**), dysfunction (**Figures 9D–F**), Merck18 (**Figures 9G–I**), MSI (**Figures 9J–L**), TAMM2 (**Figures 9M–O**).

### GMLncSigs for Tumor Chemotherapy Drug Screening

**Figure 10:**
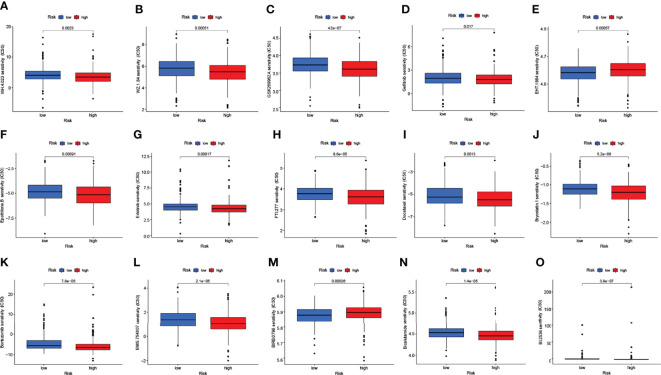
The chart showed the top 15 potential drugs for NSCLC. Respectively, WH.4 **(A)**, WZ.1.84 **(B)**, GSK269962A **(C)**, Gefitinib **(D)**, EHT.1864 **(E)**, Epothilone.B **(F)**, Erlotinib **(G)**, FTI.277 **(H)**, Docetaxel **(I)**, Bryostatin.1 **(J)**, Bortezomib **(K)**, BMS.754807 **(L)**, BIRB.0796 **(M)**, Bicalutamide **(N)**, BI.2536 **(O)**.

To identify potential drugs targeting our lncRNA model for the treatment, we used the pRRophetic algorithm to estimate the therapeutic response of the samples, which was based on the half-maximal inhibitory concentrations (IC50) available in the GDSC database. We found that the algorithm screened a total of 58 compounds and most of them had significantly different estimated half-inhibitory concentrations between the high and low subgroups, with the high-risk subgroup being more sensitive to most of them. The figure below shows the top 15 compounds that could potentially be used in NSCLC patients (**Figures 10A–O**).

### To Verify With the IMvigor210 Immunotherapy Model

**Figure 11:**
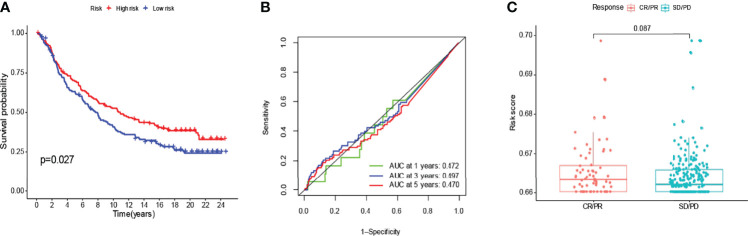
To verify with the IMvigor210 immunotherapy model. In the IMvigor210 model, high-risk scoring survival was higher **(A)**. Poor prediction of ROC curves **(B)**. No significance between responses to different drugs for immunotherapy **(C)**.

We compared the genes obtained from the LASSO regression analysis. They are LINC00707, EMS2OS, and NR2F2-AS1. To validate the prognostic value of the relevant lncRNAs, we calculated the corresponding risk score for each patient in the IMvigor210 cohort by formula and classified the patients into high and low-risk subgroups. It can be seen that there was a significant difference in survival probability between high and low-risk groups in the expression of the target gene in IMvigor210 bladder cancer (p=0.027) (**Figure 11A**), and then we validated this model using ROC curves, which unfortunately did not predict well (**Figure 11B**). The risk score of target genes was not significant between different drug responses to immunotherapy for MVigor210 bladder cancer (p=0.081) (**Figure 11C**).

### Analysis of Stemness Indices in NSCLC

**Figure 12:**
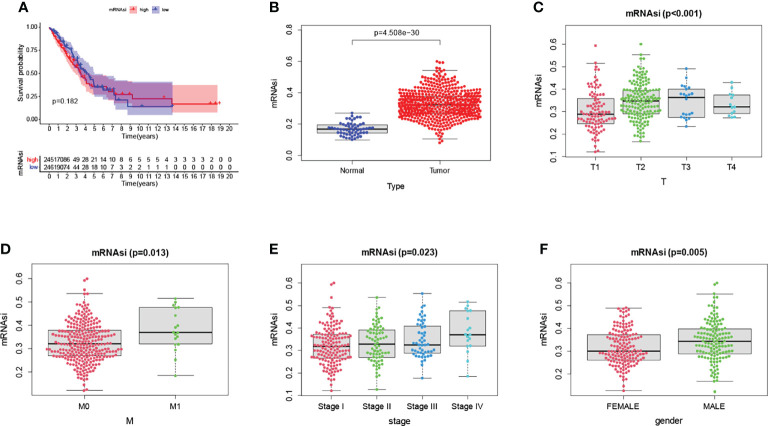
There was no difference in overall survival between the various risk groups. **(A)**. The mRNAsi was significantly different between normal and tumor tissues **(B)**. Significant differences in mRNAsi between clinicopathological factors **(C–F)**.

The mRNAsi is a calculated index based on gene expression data. Unfortunately, we did not find a correlation between mRNAsi and OS among high and low-risk subgroups (p=0.182) (**Figure 12A**). Immediately afterward, we compared the indices of normal and tumor tissues and found a significant correlation between them (**Figure 12B**). We also analyzed the mRNAsi and clinical correlations, including T, M, stage, and gender (**Figures 12C–F**). The mRNAsi was higher in males than in females. The mRNAsi increased with increasing stage.

## Discussion

In recent years, along with the rapid development of bioinformatics, many reliable prognostic signatures based on lncRNAs have been discovered to determine the prognosis of patients with various malignancies (58–65). For example, one study systematically elaborated on the relationship between m6A RNA methylation regulators and uterine cancer. They constructed a risk profile capable of predicting the prognosis of uterine cancer (65). Studies have also demonstrated the importance and value of lncRNA in the assessment of tumor immune infiltration and highlighted the potential of lncRNA combined with specific immune checkpoint factors as predictive biomarkers of ICI response, allowing for more accurate patient selection (66). In this study, we used gefitinib metabolic lncRNAs-related to establish a comprehensive algorithm to systematically evaluate NSCLC patients, including prognostic signature, TME, and drug resistance analysis.

In this study, we confirmed the importance of gefitinib metabolism-related lncRNA evaluation in NSCLC patients. Firstly, in the training cohort, 13 lncRNAs were identified for prognostic risk signature, including: NR2F2-AS1, AC092168.2, AC004884.2, AC090236.2, AL589986.2, AC026355.2, AC243772.2, AL133445.2, AC009268.2, AC090617.5, AC032011.1, AC007686.2, and LINC01754. These genes were applied to the survival analysis of the training group. The data showed that the overall survival of NSCLC patients in the high-risk subgroup was significantly lower than that of NSCLC patients in the low-risk subgroup. We also found AL133445.2, LINC01754, and AC090236.2 to be lowly expressed in the high-risk subgroup and highly expressed in the low-risk subgroup, indicating that they are protective genes. Subsequently, we confirmed the previously mentioned results in the testing cohort and the entire cohort. In our analysis of the relationship between risk scores and clinical factors, we found that early-stage tumors were significantly associated with the low-risk subgroup, while late-stage tumors were significantly associated with the high-risk subgroup. We explored the relationship between patient clinicopathological factors and OS, and univariate regression analysis showed that AJCC stage, T stage, N stage, and risk score were significantly associated with OS while multivariate regression analysis showed that only risk score was significantly associated with OS. The ROC curves suggested that clinicopathological factors were significant predictors of patient survival at 1-years, 3-years, and 5-years, and then we compared the predictive effect of risk characteristics and clinicopathological factors, and risk characteristics were better predictors than clinicopathological factors. The concordance index validated the results of the ROC curve. We chose the nomogram to visualize the prediction model, while the calibration plots of 1-years, 5-years, and 10-years OS rates were well predicted compared to the ideal model. GO and KEGG pathway enrichment analyses further validated that gefitinib metabolism is related to cytochrome P450 (hsa00982: Drug metabolism-cytochrome P450, hsa00980: Metabolism of xenobiotinib and cytochrome P450) (14). In addition, it was verified that gefitinib is associated with lipid metabolism (hsa04975: Fat digestion and absorption, hsa00592: alpha-Linolenic acid metabolism). Lipid-rich diets can promote tumor development and this class of drugs may act as nutritional modifiers in the future to improve the average survival of cancer patients (67). Serum total cholesterol, HDL, and LDL cholesterol levels were significantly reduced in gefitinib-treated mice (68). Inhibition by modulation of EGFR provides new insights into the development of drugs for the treatment of hypercholesterolemia.

The risk model was significantly associated with multiple immune microenvironment features. Type_II_IFN_Reponse, HLA and T_cell_co-stimulation are a group of low-risk pathways. Such findings were also found in the breast cancer model with iron droop-associated lncRNAs (69). In the tumor mutation burden analysis, we found no statistically significant TMB between the high-risk and low-risk subgroups. We concluded that the TIDE composite score was not significant between the high-risk and low-risk subgroups, while MDSC, CAF expression, and risk score were significantly and positively correlated. Yu *et al.* revealed a significant positive correlation between PUDP and tumor immune cell infiltration, immune cell biomarkers, and immune checkpoint expression, particularly with pro-tumor immune cells such as T cell regulatory (Treg), MDSC, and CAF (70). Next, we identified compounds that might be used to treat patients with NSCLC, and anticancer therapy with various drugs might be indicated for patients with higher risk scores. LINC00707, EMS2OS, and NR2F2-AS1 were expressed in IMvigor210 bladder cancer, where a high-risk score implies a higher survival rate, which was unexpected. Increased expression of stem cell-associated biomarkers in tumor cells is highly correlated with drug resistance, cancer recurrence, and tumor proliferation (71, 72). In our study, the stem cell index was not significantly associated with OS between high and low expression subgroups, suggesting that the degree of cancer progression is not related to OS. Some findings showed that risk scores of certain biomarkers were significantly and positively correlated with DNAss and RNAss (73, 74). Significant differences in clinicopathological factors and mRNAsi validated the reliability of the nomogram in predicting patient prognosis.

Nevertheless, our study has some limitations. First, the construction and evaluation of our prognostic prediction model were based on existing data in public databases. Therefore, further experimental and clinical studies are needed to validate our findings. Second, our study failed to identify the specific signaling pathways that regulate the growth and progression of NSCLC.

Overall, 13 gefitinib metabolic lncRNA-related were identified for the construction of prognostic models for NSCLC patients. Risk signature can accurately predict the OS of patients. GO and KEGG pathway enrichment analysis further illustrated that gefitinib metabolism is associated with cytochrome P450 and lipid metabolism. In immune-related analysis, Type_II_IFN_Reponse, HLA, and T_cell_co-stimulation are a set of protective pathways. TMB is associated with survival in NSCLC patients. Expression of MDSC, exclusion, CAF, and risk score are significantly and positively correlated. Target gene expression in IMvigor210 bladder cancer showed a significant difference in the probability of survival between high-risk and low-risk subgroups. The degree of cancer progression was not related to the risk subgroup. Finally, drugs that might be used to treat NSCLC patients were screened.

## Data Availability Statement

The original contributions presented in the study are included in the article/**Supplementary Material**. Further inquiries can be directed to the corresponding authors.

## Ethics Statement

The studies involving human participants were reviewed and approved by The work was approved by the Guangdong Medical University ethics committee. Written informed consent for participation was not required for this study in accordance with the national legislation and the institutional requirements.

## Author Contributions

XZ and MZ conceived the work. WY studied and drafted the manuscript. ZW, PG, JK, YX, CW, MZ, and XZ discussed the manuscript. ZW, MZ, and XZ edited the manuscript. XZ checked the statistical and bioinformatic accuracy as an expert in statistics and bioinformatics. All authors read and approved the final version of the manuscript.

## Funding

This work was supported partly by the National Natural Science Foundation of China (81541153); and the Graduate Innovation Experiment Project of Guangdong Medical University (ZYDS001-2021).

## Conflict of Interest

The authors declare that the research was conducted in the absence of any commercial or financial relationships that could be construed as a potential conflict of interest.

## Publisher’s Note

All claims expressed in this article are solely those of the authors and do not necessarily represent those of their affiliated organizations, or those of the publisher, the editors and the reviewers. Any product that may be evaluated in this article, or claim that may be made by its manufacturer, is not guaranteed or endorsed by the publisher.
