# Supplementary material for: Identified Gefitinib Metabolism-Related lncRNAs can be Applied to Predict Prognosis, Tumor Microenvironment, and Drug Sensitivity in Non-Small Cell Lung Cancer
Source: Front Oncol. 2022 Jul 1;12:939021. doi: 10.3389/fonc.2022.939021 (PMC9376789; doi:10.3389/fonc.2022.939021)
Supplement: Supplementary file 1 [file DataSheet_1.pdf]

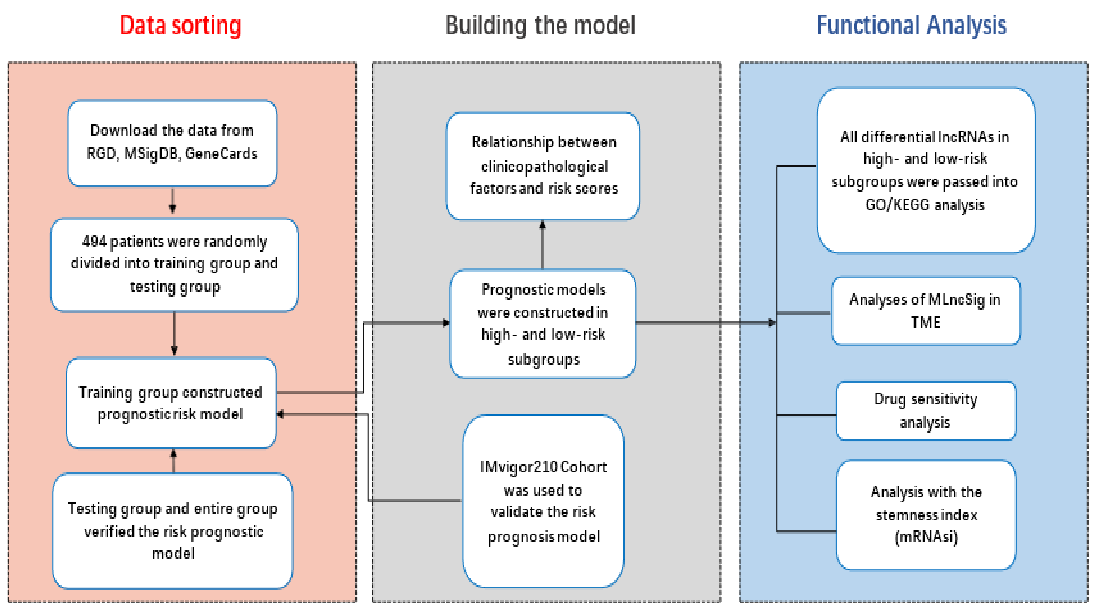

Figure S1. The flowchart in this study.

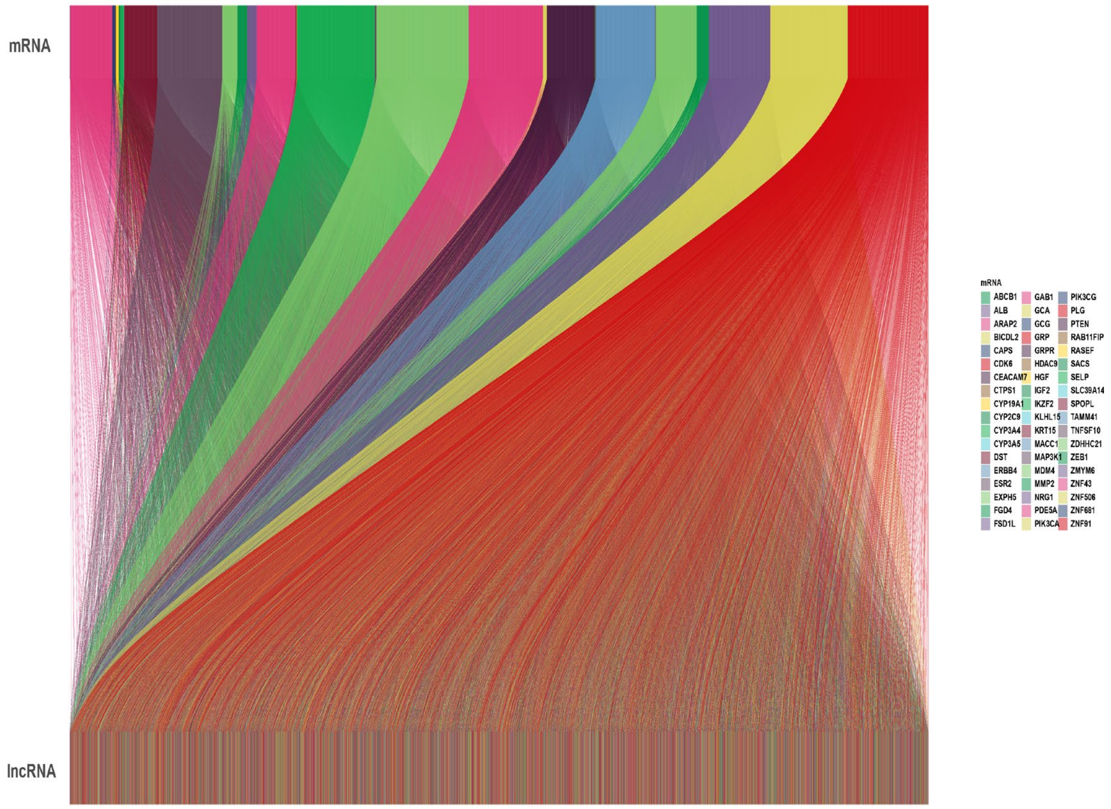

Figure S2. Visualization of target genes and co-expressed lncRNAs. The top right panel shows the lncRNAs that are significantly co-expressed with the target genes.

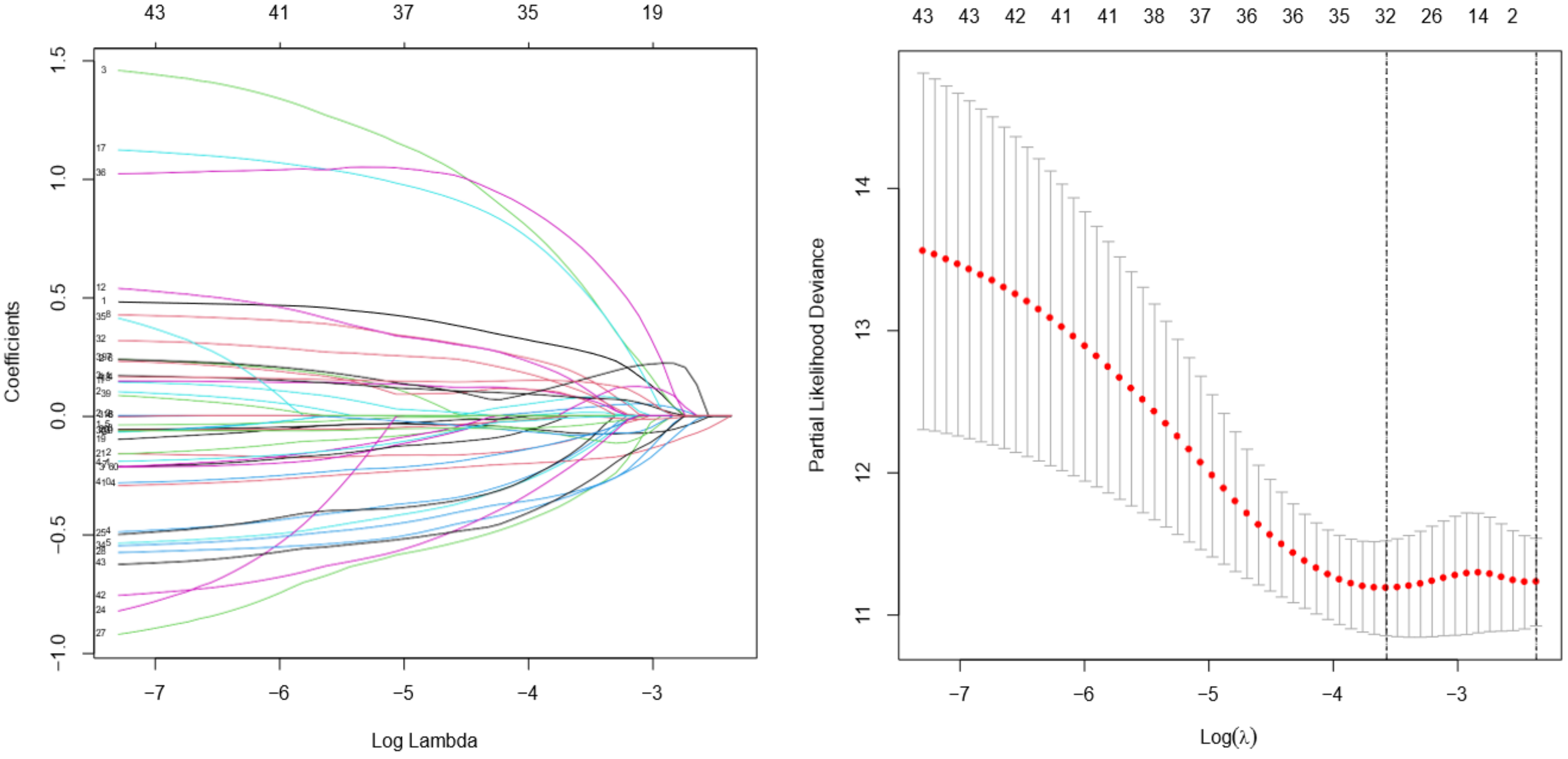

43 41 37 35 19
Coefficients
1.5
1.0
0.5
0.0
−0.5
−1.0
−7 −6 −5 −4 −3
Log Lambda
43 43 42 41 41 38 37 36 36 35 32 26 14 2
Partial Likelihood Deviance
14
13
12
11
−7 −6 −5 −4 −3
Log(λ)

A

B

Figure S3. Further identification of target genes using lasso Cox regression analysis. In the training cohort (n=330), the determination of the best λ was attained when the minimum value of the partial likelihood deviation was reached, and the Lasso coefficient for the most useful prognostic gene was further generated (A). Results are presented as a mean ± 95% confidence interval [CI] and show the identification of the 32 GMLncSigs (B).

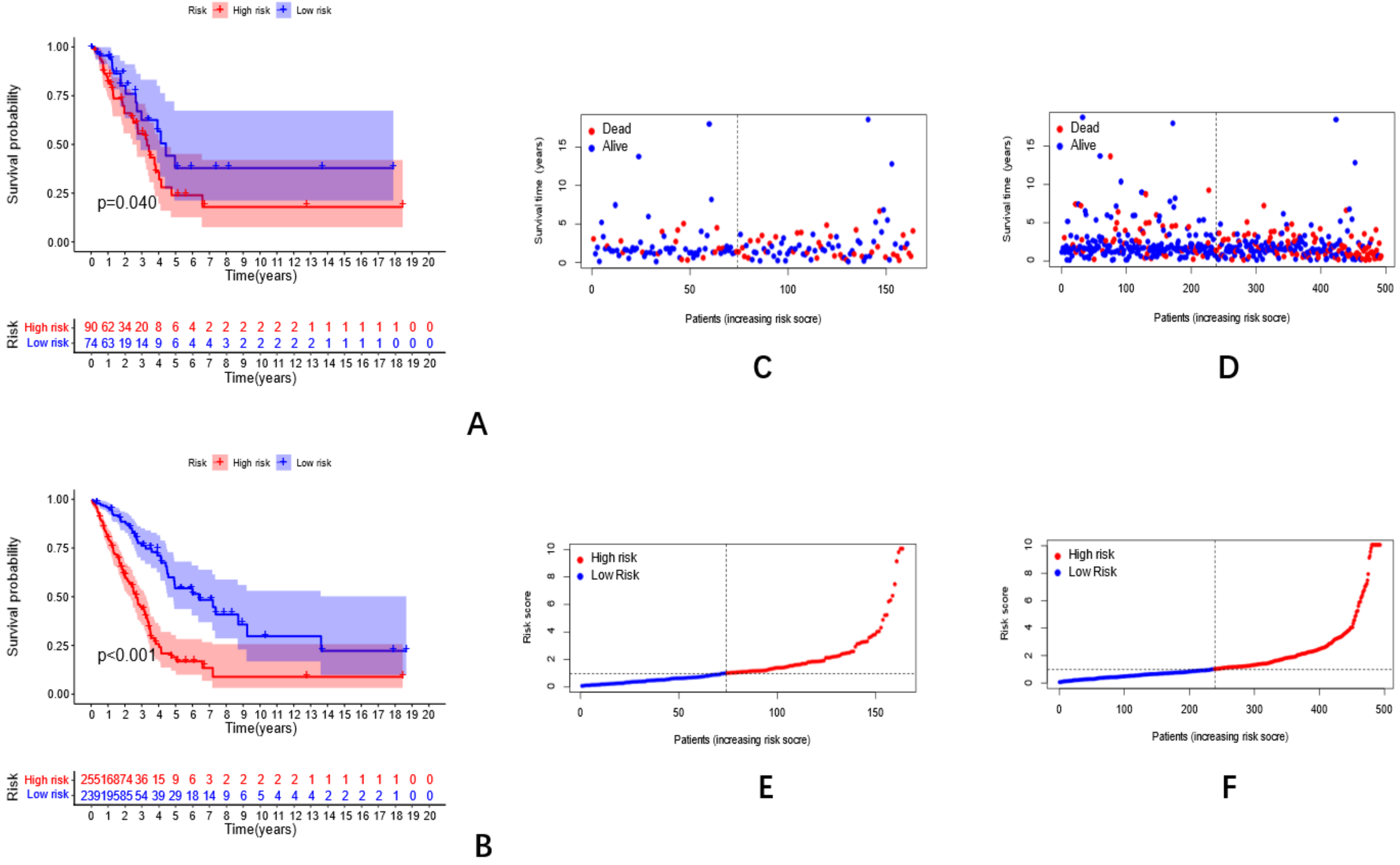

Risk High risk Low risk
Survival probability
1.00
0.75
0.50
0.25
0.00
p=0.040
Time(years)
Risk
High risk 90 62 34 20 8 6 4 2 2 2 2 2 2 1 1 1 1 1 1 0 0
Low risk 74 63 19 14 9 6 4 4 3 2 2 2 2 2 1 1 1 1 0 0 0
0 1 2 3 4 5 6 7 8 9 10 11 12 13 14 15 16 17 18 19 20
Time(years)
A
Risk High risk Low risk
Survival probability
p<0.001
Time(years)
Risk
High risk 255168 74 36 15 9 6 3 2 2 2 2 2 1 1 1 1 1 1 0 0
Low risk 239195 85 54 39 29 18 14 9 6 5 4 4 4 2 2 2 2 1 0 0
Time(years)
B
Dead
Alive
Survival time (years)
Patients (increasing risk socre)
C
Dead
Alive
Survival time (years)
Patients (increasing risk socre)
D
High risk
Low Risk
Risk score
Patients (increasing risk socre)
E
High risk
Low Risk
Risk score
Patients (increasing risk socre)
F

Figure S4. Validation of 13-lncRNA signature in the testing cohort and entire cohort. (A), (C), and (E) are the overall survival curves, distribution of risk scores, and survival status in the testing cohort, respectively. (B), (D), and (F) are the overall survival curves, distribution of risk scores, and, survival status in the entire cohort, respectively.

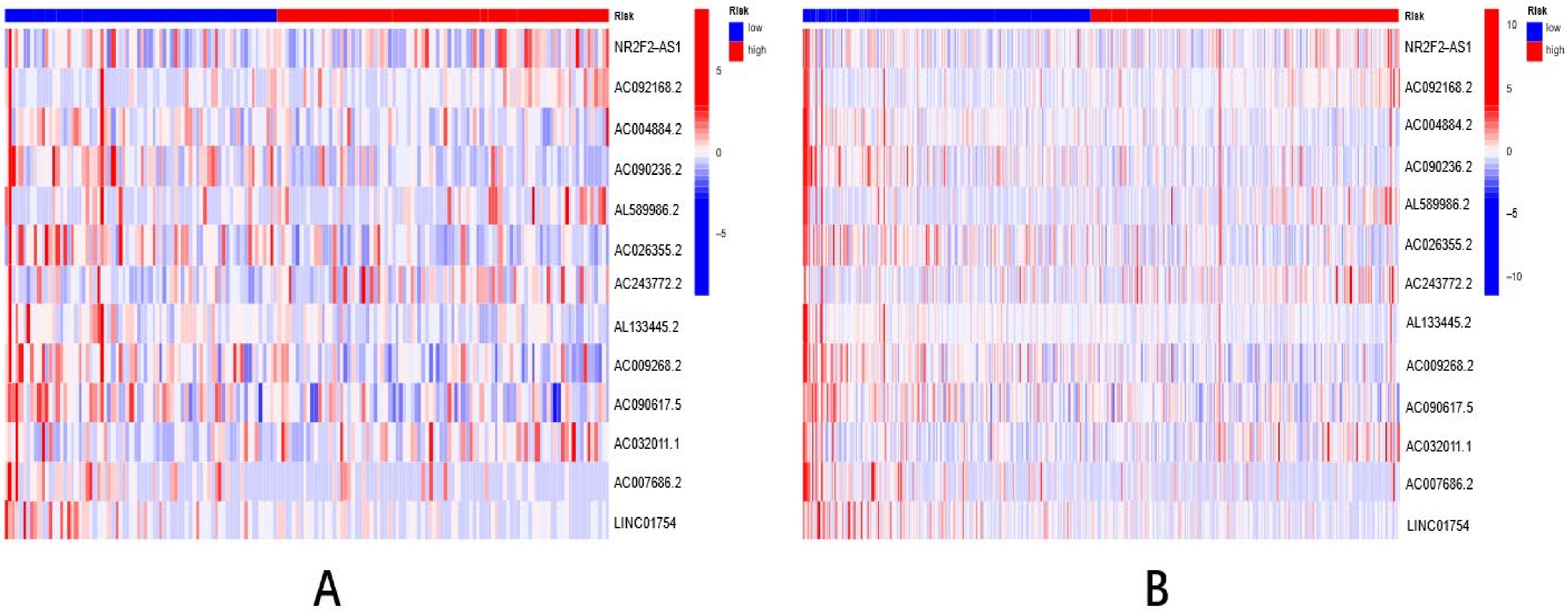

Figure S5. Heatmap of the expression profile in the 13 GMLncSigs. Testing cohort (A). Entire cohort (B).

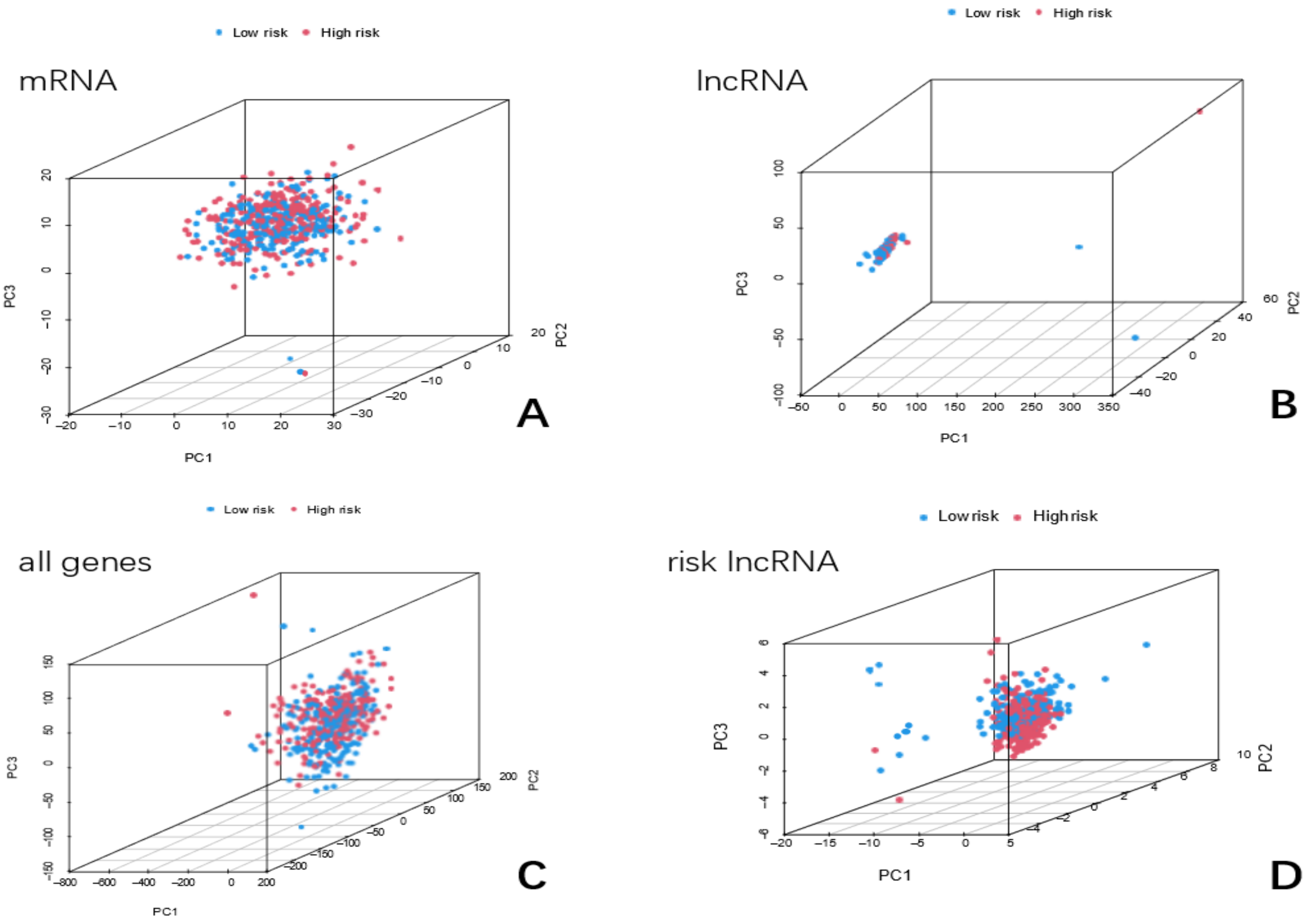
Low risk
High risk
mRNA
PC1
PC2
PC3
A
Low risk
High risk
lncRNA
PC1
PC2
PC3
B
Low risk
High risk
all genes
PC1
PC2
PC3
C
Low risk
High risk
risk lncRNA
PC1
PC2
PC3
D

Figure S6. Principal component analysis. lt revealed gene-expression differences between high- and low-risk subgroups in mRNA (A), lncRNA (B), all genes (C), and risk lncRNA (D).

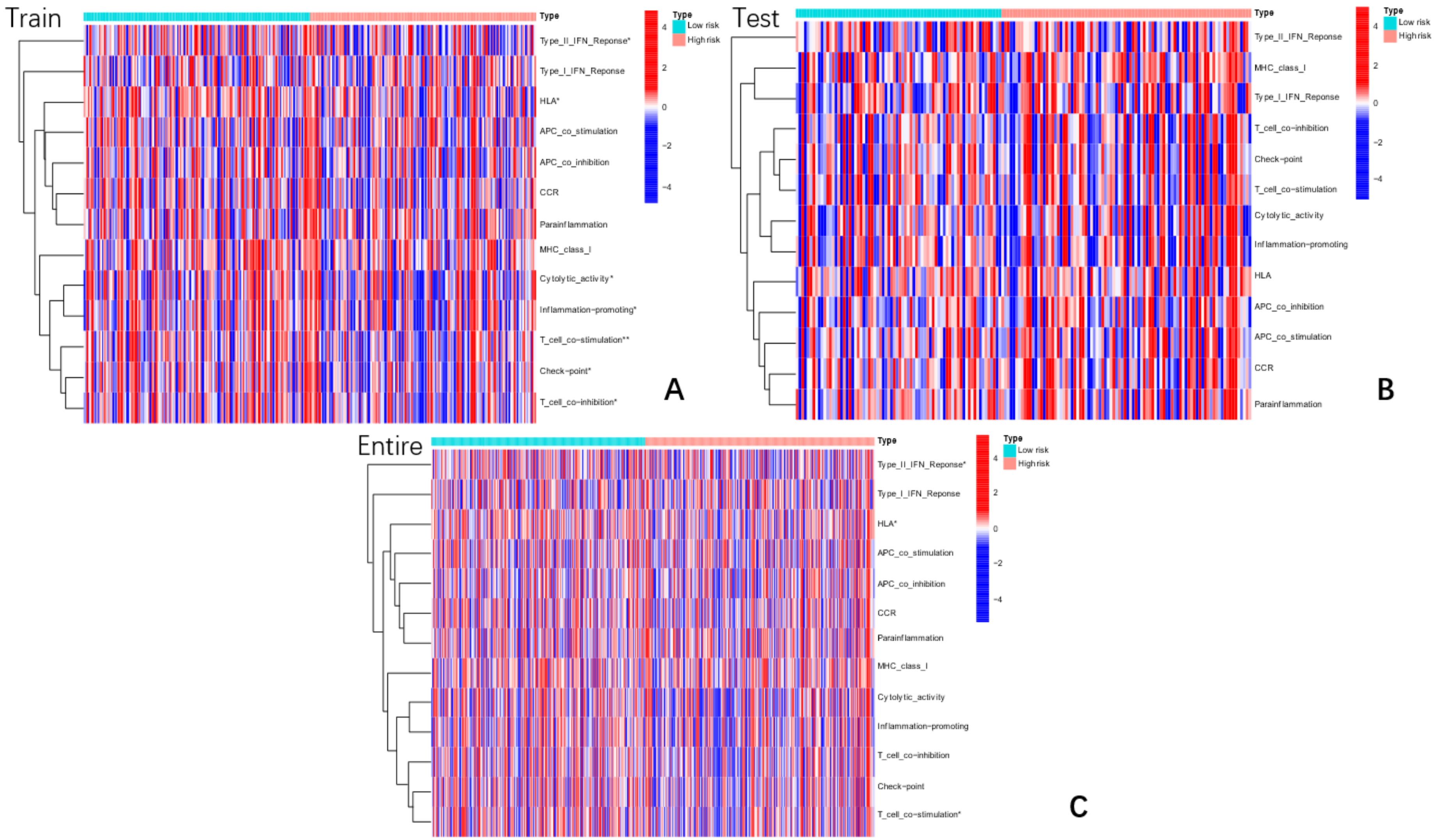
Train
Type
Type_II_IFN_Reponse*
Type_I_IFN_Reponse
HLA*
APC_co_stimulation
APC_co_inhibition
CCR
Parainflammation
MHC_class_I
Cytolytic_activity*
Inflammation−promoting*
T_cell_co−stimulation**
Check−point*
T_cell_co−inhibition*
4
2
0
−2
−4
Type
Low risk
High risk
A
Test
Type
Type_II_IFN_Reponse
MHC_class_I
Type_I_IFN_Reponse
T_cell_co−inhibition
Check−point
T_cell_co−stimulation
Cytolytic_activity
Inflammation−promoting
HLA
APC_co_inhibition
APC_co_stimulation
CCR
Parainflammation
4
2
0
−2
−4
Type
Low risk
High risk
B
Entire
Type
Type_II_IFN_Reponse*
Type_I_IFN_Reponse
HLA*
APC_co_stimulation
APC_co_inhibition
CCR
Parainflammation
MHC_class_I
Cytolytic_activity
Inflammation−promoting
T_cell_co−inhibition
Check−point
T_cell_co−stimulation*
4
2
0
−2
−4
Type
Low risk
High risk
C

Figure S7. Comparison of 13 immune-related pathway enrichments between high-risk and low-risk subgroups. In the training cohort (A). In the testing cohort (B). In the entire cohort (C).

gene	HR	HR.95L	HR.95H	pvalue
NR2F2-AS1	1.306599356	1.048744571	1.627852885	0.017111935
AC008073.2	0.407311154	0.169054945	0.981351808	0.045293251
AC090236.2	0.662104917	0.449126354	0.976079263	0.037321518
AL135960.1	0.233303923	0.06776693	0.803204761	0.021032821
AL021026.1	0.573753614	0.335903377	0.980023521	0.041968097
SH3BP5-AS1	0.951126208	0.912470271	0.991419767	0.017931681
AP000302.1	0.380789585	0.159933577	0.906630807	0.029151933
AP002026.1	0.787341291	0.650801143	0.952527997	0.013875688
AC006017.1	0.851433952	0.740034021	0.979603308	0.024576173
AC015914.1	0.793748458	0.633643571	0.994307594	0.044470351
AC087854.1	0.691122933	0.485657119	0.983514687	0.040139858
AL353801.3	0.731647996	0.559237865	0.957211276	0.022671792
AC026462.3	1.093499334	1.027903565	1.163281103	0.004626879
AL109811.1	0.512169913	0.28061647	0.934791959	0.029285274
AC010999.2	0.562749926	0.367079407	0.862721998	0.008355905
AC021086.1	2.481531523	1.192793485	5.162669627	0.015030732
PRR34	1.467169103	1.027510304	2.0949524	0.034919188
AF131215.5	0.911574537	0.840305868	0.9888877	0.025813824
AC009318.2	1.082688727	1.015125777	1.154748412	0.015665776
ADPGK-AS1	0.297316148	0.109276019	0.80893221	0.017540705
AC009318.3	1.106497465	1.016513364	1.204447165	0.019365336
AC005291.1	1.039277216	1.011435646	1.067885174	0.005424553
AC026355.2	0.916802154	0.870389078	0.965690184	0.001048816
AC090948.1	0.865076447	0.762808529	0.98105518	0.023950003
LINC00239	1.024292103	1.005320704	1.043621511	0.011859489
AC105036.3	1.269835586	1.027776973	1.568903038	0.026837373
AC079949.1	1.010203792	1.000286106	1.020219812	0.043717118
AC108134.4	0.931326444	0.867690202	0.999629758	0.048813283
AL359878.1	0.712798689	0.521658622	0.973974071	0.033542924
AL021368.2	0.761648876	0.580252637	0.999752476	0.049791871
FOCAD-AS1	0.666813592	0.450880406	0.986160324	0.042380174
AP003064.2	0.795293091	0.642010146	0.98517306	0.036019406
AC012676.4	0.661909175	0.455714996	0.961398591	0.030259806
AC034102.8	0.75289254	0.602980774	0.940075043	0.012229738
LINC01600	1.457417177	1.057064035	2.009400337	0.021526096
AL121985.1	0.455818791	0.207952639	0.999125431	0.049745124
AL356608.1	0.255017943	0.070854663	0.917852802	0.036515016
AC114550.2	0.537777959	0.290685719	0.994906576	0.048128451
AC004704.1	1.020940511	1.009200998	1.032816583	0.000444555
LINC02410	0.509924384	0.260126444	0.99960186	0.049864612
AL031600.2	0.450346933	0.253046479	0.801482642	0.006680274
AL162632.3	3.695901635	1.69840779	8.042643809	0.000983583
AL138826.1	1.081918195	1.003449042	1.166523592	0.040404193
GLCCI1-DT	0.661247844	0.447525873	0.977035604	0.037838445
LINC00707	1.021070557	1.012762422	1.029446847	5.67E-07
AP000977.1	0.291992556	0.092806311	0.918683784	0.035292162
AC026356.1	1.066578976	1.003872934	1.133201895	0.037069134
AP001094.3	1.225739043	1.005455273	1.494284471	0.044031609
AC090617.5	0.907419292	0.8395815	0.980738346	0.014263096
AC112721.2	1.046334102	1.012593087	1.081199415	0.006763683
AL161668.1	1.026161423	1.012255933	1.040257935	0.000207359
ZEB2-AS1	0.445124159	0.206371132	0.960093184	0.039036309
AL137779.1	0.649380947	0.441341573	0.955485819	0.028447772
LINC02147	0.100883916	0.021672901	0.469598633	0.003463647
AC007686.2	0.242396846	0.075328008	0.780005111	0.017471814
TMEM18-DT	0.261411461	0.070913459	0.963652778	0.043843307

Table S1. Results of the univariate Cox regression analysis of the entire cohort. Univariate Cox regression analysis results show the p values and hazard ratios (HR) with confidence intervals (CI) of the 56 differentially expressed genes in the entire cohort.

id	HR	HR.95L	HR.95H	pvalue
NR2F2-AS1	1.595470646	1.023724933	2.486533737	0.03906175
AC009970.1	0.438344087	0.224521586	0.855799845	0.015685601
AC092168.2	1.424915126	1.097449274	1.850092905	0.007862312
AC004884.2	0.66904836	0.447834116	0.999534632	0.049734941
AC090236.2	0.565910487	0.326222754	0.981705522	0.042799722
AL078645.1	0.556602864	0.319442909	0.969834482	0.038632591
IGBP1-AS2	0.443518537	0.20088748	0.97919837	0.044221505
AL589986.2	1.694587002	1.085174989	2.6462323	0.020372133
AC135507.1	0.617769873	0.39489458	0.966434173	0.034901383
AC087854.1	0.501519863	0.282830692	0.889302965	0.018206317
AC084757.2	2.060638376	1.052247444	4.035391619	0.03498925
AC103923.1	1.881866648	1.011106134	3.502522593	0.046063498
AF131215.5	0.708339797	0.555426579	0.903351204	0.00544996
AC026355.2	0.698581518	0.565733487	0.862625509	0.000858855
AC090948.1	0.667122662	0.487967098	0.912054621	0.011183644
AC079949.1	1.249624842	1.061483977	1.471112405	0.007436041
AC243772.2	1.700182901	1.0185748	2.837908319	0.042318798
AC127164.1	1.507055755	1.013905384	2.240068042	0.042534417
AL021368.2	0.617374947	0.392888728	0.970126652	0.036484189
AC093278.2	0.74690765	0.567863722	0.982403024	0.036894401
AC027097.2	0.554546965	0.338700338	0.907948121	0.019085809
AC034102.8	0.637363542	0.440273409	0.922681853	0.017018951
EMX2OS	1.744453164	1.175062831	2.589748192	0.005776791
LINC01600	2.32969225	1.291561536	4.202251171	0.004953162
AL133445.2	0.363178146	0.17762829	0.742552699	0.005508514
AC083806.3	1.622780707	1.000457639	2.63221262	0.049783679
AL136456.1	0.207717576	0.053003426	0.814034006	0.024119461
AC009268.2	0.561357703	0.359761388	0.875920767	0.010972684
AC012181.1	0.709874086	0.533595047	0.94438886	0.018631145
AC004704.1	1.251125344	1.06736667	1.466520054	0.005703282
LINC00707	1.260296197	1.064970976	1.491445815	0.007089779
AC012409.4	1.641708007	1.015856299	2.653136258	0.042949506
AC073534.2	0.750900638	0.569838391	0.989494175	0.041852336
AC090617.5	0.555778659	0.368090216	0.839169054	0.005205726
PIK3CD-AS1	0.201317872	0.052906655	0.766045126	0.018730757
AC032011.1	2.51959382	1.018690874	6.231873854	0.045495554
AL161668.1	1.743700968	1.356126962	2.242041601	1.46E-05
ELN-AS1	0.837389278	0.719658327	0.974380169	0.021692972
AC016727.1	0.686432163	0.471321294	0.999719554	0.049829328
AL137779.1	0.481225844	0.277071546	0.835806911	0.009411542
AC074032.1	0.671718805	0.452346284	0.997479516	0.04855554
AC007686.2	0.291708398	0.087566534	0.971761542	0.044790224
LINC01754	0.481020137	0.239651448	0.96548706	0.039516481
AC090116.1	1.496815927	1.04873132	2.136350728	0.026276523

Table S2. Results of the univariate Cox regression analysis of the training cohort. Univariate Cox regression analysis results show the p values and hazard ratios (HR) with confidence intervals (CI) of the 44 differentially expressed genes in the training cohort.

id	coef	HR	HR.95L	HR.95H	pvalue
`NR2F2-AS1`	0.499206893	1.647414177	0.975805432	2.781264977	0.061720062
AC092168.2	1.377715472	3.965831218	2.447492726	6.426093562	2.21E-08
AC004884.2	-0.521604815	0.593567218	0.390955732	0.901181421	0.014350921
AC090236.2	-0.652403679	0.520792453	0.280687474	0.966287434	0.038573625
AL589986.2	0.687458446	1.988654832	1.110930199	3.559852854	0.020663729
AC026355.2	-0.246691057	0.781382058	0.620440742	0.984071289	0.036045102
AC243772.2	1.09634434	2.993203864	1.761098759	5.087317974	5.10E-05
AL133445.2	-0.798432481	0.450033848	0.248061659	0.816452107	0.008608258
AC009268.2	-0.598224282	0.549787036	0.327803553	0.922094293	0.023367204
AC090617.5	-0.438251398	0.64516357	0.414992413	1.002996726	0.051573986
AC032011.1	1.431682937	4.185737602	1.477250779	11.86013879	0.007055024
AC007686.2	-1.154177308	0.315316839	0.100039356	0.993855946	0.048782713
LINC01754	-0.871927311	0.418144877	0.193461002	0.903774592	0.026606348

Table S3. The results of multivariate Cox regression. It showed that the p-values and hazard ratios of 13 differentially expressed GMLncSigs had confidence intervals, ultimately. Specific data for the risk score were also included.
